# Identification of positive and negative regulators in the stepwise developmental progression towards infectivity in *Trypanosoma brucei*

**DOI:** 10.1038/s41598-021-85225-2

**Published:** 2021-03-11

**Authors:** Justin Y. Toh, Agathe Nkouawa, Saúl Rojas Sánchez, Huafang Shi, Nikolay G. Kolev, Christian Tschudi

**Affiliations:** 1 Department of Epidemiology of Microbial Diseases, Yale School of Public Health, New Haven, CT 06536 USA; 2 Department of Epidemiology of Microbial Diseases, Boyer Center for Molecular Medicine 136C, Yale School of Public Health, 295 Congress Avenue, New Haven, CT 06536 USA

**Keywords:** Parasitology, Pathogens

## Abstract

*Trypanosoma brucei* is a protozoan parasite that causes important human and livestock diseases in sub-Saharan Africa. By overexpressing a single RNA-binding protein, RBP6, in non-infectious procyclics trypanosomes, we previously recapitulated in vitro the events occurring in the tsetse fly vector, namely the development of epimastigotes and infectious, quiescent metacyclic parasites. To identify genes involved in this developmental progression, we individually targeted 86 transcripts by RNAi in the RBP6 overexpression cell line and assessed the loss-of-function phenotypes on repositioning the kinetoplast, an organelle that contains the mitochondrial genome, the expression of BARP or brucei alanine rich protein, a marker for epimastigotes, and metacyclic variant surface glycoprotein. This screen identified 22 genes that positively or negatively regulate the stepwise progression towards infectivity at different stages. Two previously uncharacterized putative nucleic acid binding proteins emerged as potent regulators, namely the cold shock domain-containing proteins CSD1 and CSD2. RNA-Seq data from a selected group of cell lines further revealed that the components of gene expression regulatory networks identified in this study affected the abundance of a subset of transcripts in very similar fashion. Finally, our data suggest a considerable overlap between the genes that regulate the formation of stumpy bloodstream form trypanosomes and the genes that govern the development of metacyclic form parasites.

## Introduction

*Trypanosoma brucei* is a protozoan parasite that causes African sleeping sickness in humans and together with *T. congolense* and *T. vivax* remains a public health concern in sub-Saharan Africa by afflicting livestock with nagana. During its complex life cycle between the mammalian host and the blood-feeding tsetse fly vector (*Glossina *sp*.*), *T. brucei* transitions through many distinct developmental forms to adapt to changing environments. In the infected mammal, *T. brucei* is coated with a variant surface glycoprotein^1^ (VSG) and exists as both a proliferative slender and a quiescent stumpy form. The transition from slender to stumpy form parasites is triggered in a cell-density dependent manner through a quorum sensing mechanism^2,3^. Stumpy form parasites are primed for survival in the tsetse fly as evident from the increased expression of mitochondrial proteins^4^, which prepares parasites to exploit oxidative phosphorylation for ATP production and use of proline as the new carbon source in the tsetse fly, and the protein associated with differentiation 1 (PAD1)^5^, which internalizes cis-aconitate and citrate that act as signaling molecules to trigger development into procyclic parasites. Upon ingestion of bloodstream form trypanosomes by the tsetse fly, the stumpy form parasites that withstand the digestive proteases^6,7^, insect immune system^8^, and alkaline conditions^9^ found in the midgut will differentiate into procyclic forms and lose mammalian infectivity^1,10^. This differentiation process is accompanied with major morphological and metabolic changes that include repositioning of the kinetoplast (an organelle that contains the mitochondrial genome) between the posterior pole and the nucleus, expansion of the mitochondrial cisternae for an oxidative phosphorylation-dependent metabolism, loss of the VSG coat, and expression of a glycoprotein coat composed mainly of procyclins rich in Gly-Pro-Glu-Glu-Thr repeats (GPEET) and procyclins rich in Glu-Pro repeats (EP)^1,11–13^.

Procyclic parasites undergo an intricate developmental process known as metacyclogenesis, whereby non-infectious trypanosomes develop into infectious metacyclic parasites, and regain mammalian infectivity through the expression of a specific subset of VSGs known as metacyclic VSGs (mVSGs). As procyclic parasites transit to a portion of the foregut in the tsetse fly known as the proventriculus, they elongate, reposition the kinetoplast anterior to the nucleus, and become epimastigotes, which undergo asymmetric division to produce long and short epimastigotes^1^. Epimastigotes travel to the salivary gland, where they express a family of alanine-rich surface proteins known as brucei alanine rich protein (BARP)^14^. A subset of these epimastigotes will attach to the epithelia of the salivary gland and gain the capacity to undergo either symmetric division to colonize the salivary gland or asymmetric division to produce infectious, quiescent metacyclic parasites^1^. Major morphological and metabolic changes occur in metacyclic parasites, which include a morphologically regressed mitochondrion that primes the parasite for a glycolysis-based metabolism^15^, increased endocytosis^16^, a repositioned kinetoplast at the posterior end of the parasite^1^, and mVSG expression^17^ for survival in the mammalian host. The molecular mechanisms underlying this developmental process remain largely unknown. Previously, this dearth of knowledge was in part due to the lack of an in vitro differentiation system, which prevented the use of large-scale genetic and biochemical experiments. This limitation was overcome with the establishment of an in vitro differentiation system, where procyclic parasites overexpressing the RNA-binding protein 6 (RBP6) progress to become metacyclic parasites^18^. This system recapitulates most of the *T. brucei* developmental processes from procyclic to metacyclic parasite and captures many of the in vivo hallmarks of differentiation^18^. Since then two additional in vitro differentiation systems have been developed. The overexpression of RBP6 with a single-point mutation (Q109K) skipped the intermediate epimastigote stage and the resulting metacyclics were able to progress to bloodstream forms in vitro^19^*.* In addition, RBP10 was shown to promote the bloodstream form differentiation state, when overexpressed in procyclic parasites^20–22^.

Among the three in vitro differentiation systems, the wild-type RBP6 overexpression system best reflects the development of trypanosomes within the tsetse fly and has opened up a better understanding of metacyclogenesis. For instance, metacyclics obtained from this system are quiescent, i.e. they are non-dividing and display a dramatic reduction in transcription, protein synthesis and the amount of ribosomal proteins^23^. Transcriptomic and proteomic analysis revealed an increase in glycolytic proteins and their corresponding mRNAs in metacyclic parasites relative to procyclic parasites, which is consistent with the progression to glycolysis as the primary mode of ATP generation^23^. Furthermore, metacyclic parasites expressing one of the eight major *T. brucei* Lister 427 mVSGs^24^ were able to establish an infection in mice^18^. Recently, mitochondrial metabolic changes in the proline oxidation pathway and tricarboxylic acid (TCA) cycle have been documented using transcriptomic, proteomic, and metabolomic approaches^25^. Upon RBP6 induction, there was an upregulation of proline catabolism and components of the TCA cycle, which fuels oxidative phosphorylation-based ATP production. Changes in the electron transport chain increased the levels of reactive oxygen species, which act as signaling molecules to drive cellular differentiation from epimastigotes to metacyclic parasites^25^. Utilizing this in vitro differentiation system, we performed a targeted RNAi screen to identify genes involved in regulating metacyclogenesis. We individually targeted 86 transcripts by RNAi in the inducible RBP6 overexpression cell line and assessed the loss-of-function effects on kinetoplast repositioning, and BARP and mVSG expression, resulting in the identification of 22 genes that positively or negatively regulate different steps of metacyclogenesis.

## Results

### RBP6 induction alters the mRNA translation landscape

**Figure 1:**
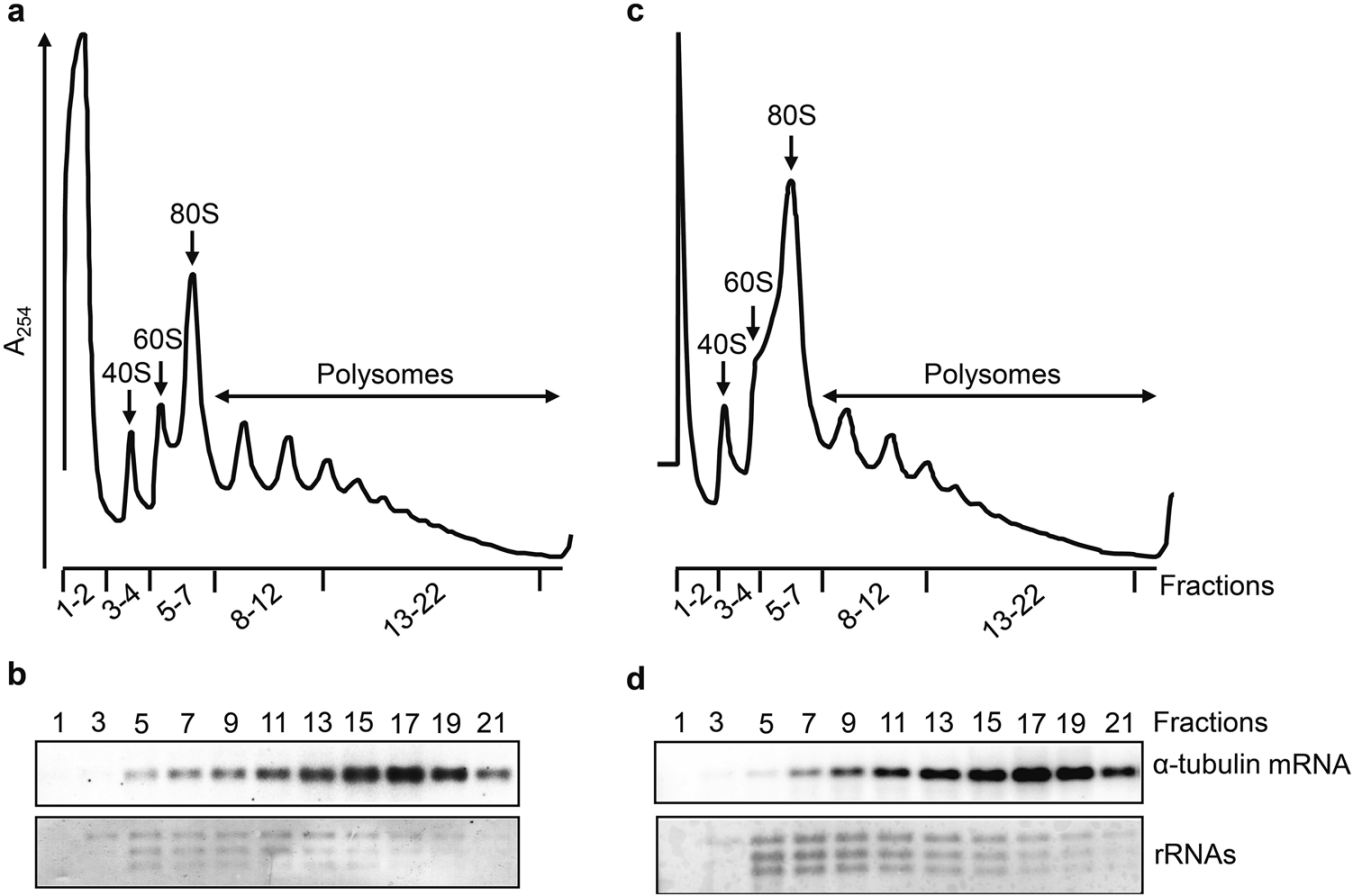
Polysome profiling. (**a**,**c**) Representative sucrose gradient absorbance profiles. Lysates obtained from un-induced (**a**) and 48 h induced (**c**) RBP6 overexpression cells were loaded on a linear 15–50% sucrose gradient and the absorbance was recorded at 254 nm. The positions of 40S, 60S, 80S and polysomes are indicated. The six pooled gradient fractions are: 1–2 (free RNA), 3–4 (40S), 5–7 (60S and 80S)), 8–12 (light polysomes), 13–22 (heavy polysomes). (**b**,**d**) Northern blot analyses for the polysomal distribution of α-tubulin mRNA of un-induced (**b**) and 48 h induced (**d**) RBP6 overexpression cells. The fraction numbers from the monosomes to the heavy polysomes that were loaded on the gel are indicated. The bottom panel in each shows the methylene blue staining of the large ribosomal RNAs in the sucrose density gradient fractions. Full-length blots of panels (**b**) and (**d**) are shown in Supplementary Fig. S9.

The goal in this project was to identify genes downstream of RBP6 that are involved in the developmental program transforming procyclic parasites into quiescent, mammalian-infectious metacyclic parasites, also referred to as metacyclogenesis. In a first set of experiments, we investigated the impact of RBP6 induction on changes in translation efficiencies, i.e. changes which are not paralleled by corresponding changes in cytoplasmic mRNA levels. Un-induced and RBP6-induced cells for 48 h were treated with the translation elongation inhibitor cycloheximide to immobilize ribosomes on mRNA and cytosolic extracts were separated on 15–50% linear sucrose density gradients by ultracentrifugation to separate polysomes from monosomes, ribosomal subunits and messenger ribonucleoprotein particles (mRNPs)^26^. This enables discrimination between efficiently translated from poorly translated mRNAs. The polysome profile derived from each sample (Fig. 1a,c) was used to determine the fractions that corresponded to mRNPs (fractions 1–2), 40S (fractions 3–4), and ribosomal subunits 60S and 80S (fractions 5–7). Using the sedimentation of α-tubulin mRNA, assayed by Northern blot (Fig. 1b,d), as a guide, we arbitrarily defined fractions 8–12 and 13–22 as light and heavy polysomes, respectively. Fractions containing heavy polysomes were pooled and along with total RNA samples analyzed by RNA-Seq (see “Methods” section). We performed three biological replicates and identified 990 and 12 transcripts whose polysome association was increased or decreased, respectively, at least threefold relative to un-induced cells (Supplementary Table S1). The abundance of 304 transcripts was upregulated at 48 h^19^ and 238 of these revealed increased polysome association. In contrast, 752 transcripts with increased polysome association did not change transcript abundance levels (Supplementary Table S1), thus highlighting translation as an important regulatory step.

### Targeted RNAi screen identifies 22 genes that play a role in metacyclogenesis

Since we know that the induction of RBP6 expression for only one day results in the generation of metacyclics (Supplementary Fig. S1), we hypothesized that RBP6 is triggering a cascade of gene expression, biochemical, and morphological events. Initially, we attempted a genome-wide RNAi screen to identify genes downstream of RBP6, but encountered technical difficulties, likely due to an apparent interference between the RNAi library and the RBP6 overexpression system. We therefore designed a targeted RNAi screen based mainly upon our available transcriptomic^19,23^ and proteomic^23^ datasets that documented differential gene expression during the transformation from procyclic to epimastigote and, subsequently, into metacyclic parasites. We primarily chose candidates that encode protein kinases, protein phosphatases, cell-cycle regulators, and DNA- and/or RNA-binding proteins, because these categories of proteins are often associated with developmental regulation in eukaryotic organisms^3,27–30^. We included 61 genes whose transcripts were at least two-fold upregulated after RBP6 overexpression for 24 h or in metacyclic parasites^19^. We also included 3 genes that increased in protein abundance by at least two-fold in metacyclic parasites relative to procyclic parasites^23^. Additionally, 8 genes whose transcripts were approximately two-fold downregulated in metacyclic parasites compared to procyclic parasites were added to the screen, since these genes could be negative regulators of metacyclic development^19^. Finally, based on the experiments described above, we included 25 genes whose transcripts increased in polysome association by at least two-fold in RBP6-induced cells for 48 h when compared to un-induced parasites (Supplementary Table S1). From this set of 97 RNAi targets, we eliminated 11 genes, whose down-regulation has been shown to cause growth arrest or parasite death by a previous genome-wide RNAi screen for procyclic parasite fitness^31^. Of the 86 total candidate genes (Supplementary Table S2), 15 genes were previously identified to play an important role in the development of bloodstream parasites from slender to stumpy forms^3^. Since both short stumpy and metacyclic parasites are quiescent and pre-adapted for transmission, we hypothesized that these genes required for stumpy bloodstream form development may also be involved in the production of metacyclics.

Because *T. brucei* mRNA degradation by the RNAi machinery is efficiently and specifically triggered by double-stranded RNA produced in vivo as a long hairpin^32,33^, we created plasmid constructs that utilize a tetracycline-inducible promoter to transcribe hairpins targeting the open reading frame. Our list included 4 gene families where individual members were targeted simultaneously, namely genes coding for Proteins Associated with Differentiation (PAD 1–8), two hexokinases (HK1-2), a gene family of protein phosphatases 1 (PP1 4–6), and a gene family of NEK kinases (NEK17 1–3). The remaining transcripts were targeted individually, resulting in a total of 74 RNAi cell lines. Each RNAi cassette was stably integrated in the non-transcribed 177-bp repeat satellite DNA of *T. brucei* Lister 427 (29–13) cells, while the tetracycline-inducible RBP6 construct was stably integrated in the non-transcribed rDNA spacer. Therefore, upon doxycycline exposure, each cell line simultaneously expressed RBP6 to trigger development from procyclic to metacyclic forms and a double-stranded RNA hairpin targeting a transcript of interest for RNAi downregulation. In this screen, we did not monitor the efficacy of RNAi depletion of the targeted transcripts, and thus, if an RNAi cell line did not display a phenotype, we cannot conclude that the targeted gene is not involved in metacyclic development. Additionally, the degree of RNAi efficiency for different mRNAs could influence the severity of the observed phenotype. Lastly, our experimental design was aimed at analyzing genes acting downstream of RBP6, so any genes operating upstream will not be detected, particularly because protein overexpression generally occurs more rapidly than RNAi in *T. brucei*.

**Figure 2:**
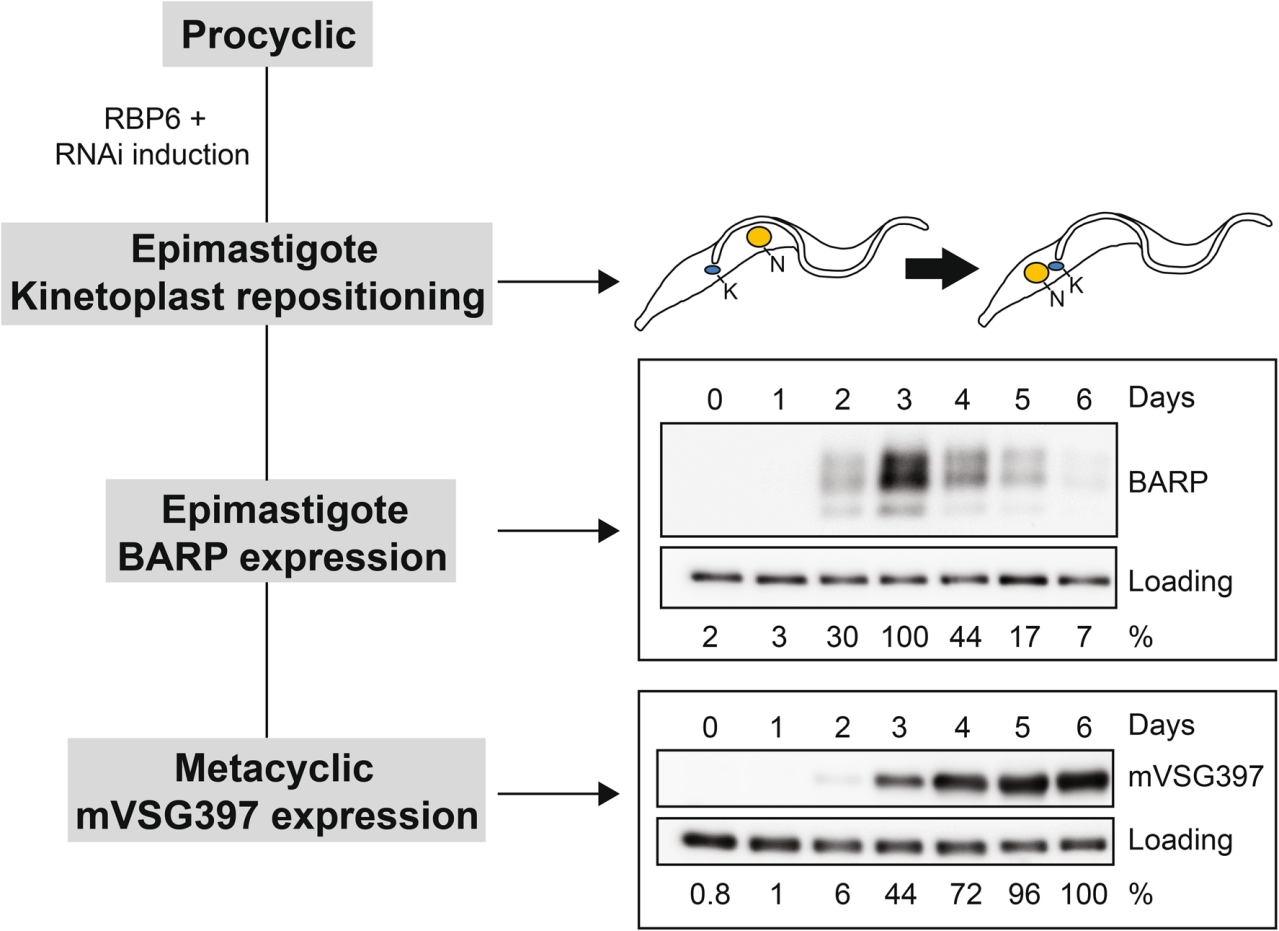
Targeted RNAi screen overview. BARP and mVSG397 are surface markers that serve as a positive readout for epimastigote and metacyclic production, respectively. Epimastigotes were quantified on the third day and metacyclics on the sixth day, since these are the times where BARP and mVSG397 expression was highest in our 6-day time course. Each RNAi cell line was induced with doxycycline to overexpress RBP6 to stimulate development and to express an RNAi hairpin that down-regulates a specific transcript of interest. After three days of induction, cells were scored as epimastigotes, if their kinetoplast localized anterior to the nucleus and these results were compared to the positive control, the wild-type RBP6 overexpression cell line. Induced cells from the targeted RNAi screen and the positive control were also harvested on the third and sixth day of doxycycline induction for Western blot analysis to quantify BARP and mVSG397 expression, respectively. Full-length blots are shown in Supplementary Fig. S10.

The RBP6 overexpression system we use will result in approximately 30% of the cells developing into epimastigotes after 2–3 days and approximately 50% of the cells will be infectious metacyclic parasites after 4–6 days. We used two experimental methods to quantify the efficacy of this developmental program (Fig. 2). First, we Hoechst stained the differentiating population and used fluorescent microscopy to distinguish and quantify epimastigote parasites based on the relative position of the parasite’s kinetoplast to its nucleus. Whereas the kinetoplast of both procyclic and metacyclic parasites is positioned posterior to the nucleus, the kinetoplast of epimastigotes is localized anterior to the nucleus. Second, we used Western blot analysis to monitor the expression of specific surface proteins. Since epimastigotes express BARP^14^ and metacyclic parasites express one of the eight major mVSGs, the expression of BARP and mVSG397, the most abundantly expressed mVSG and present on approximately 50% of metacyclics^19,34^, served as a positive readout for the quantity of epimastigote and metacyclic parasites, respectively. Taken together, Fig. 2 illustrates our assays in a 6-day time course of RBP6 overexpression in procyclic parasites. The first event to happen is the repositioning of the kinetoplast and this occurs before BARP expression, which peaks on the third day. Finally, mVSG397 expression is highest on the 6^th^ day, which correlates with the morphological counts of metacyclics^34^.

**Figure 3:**
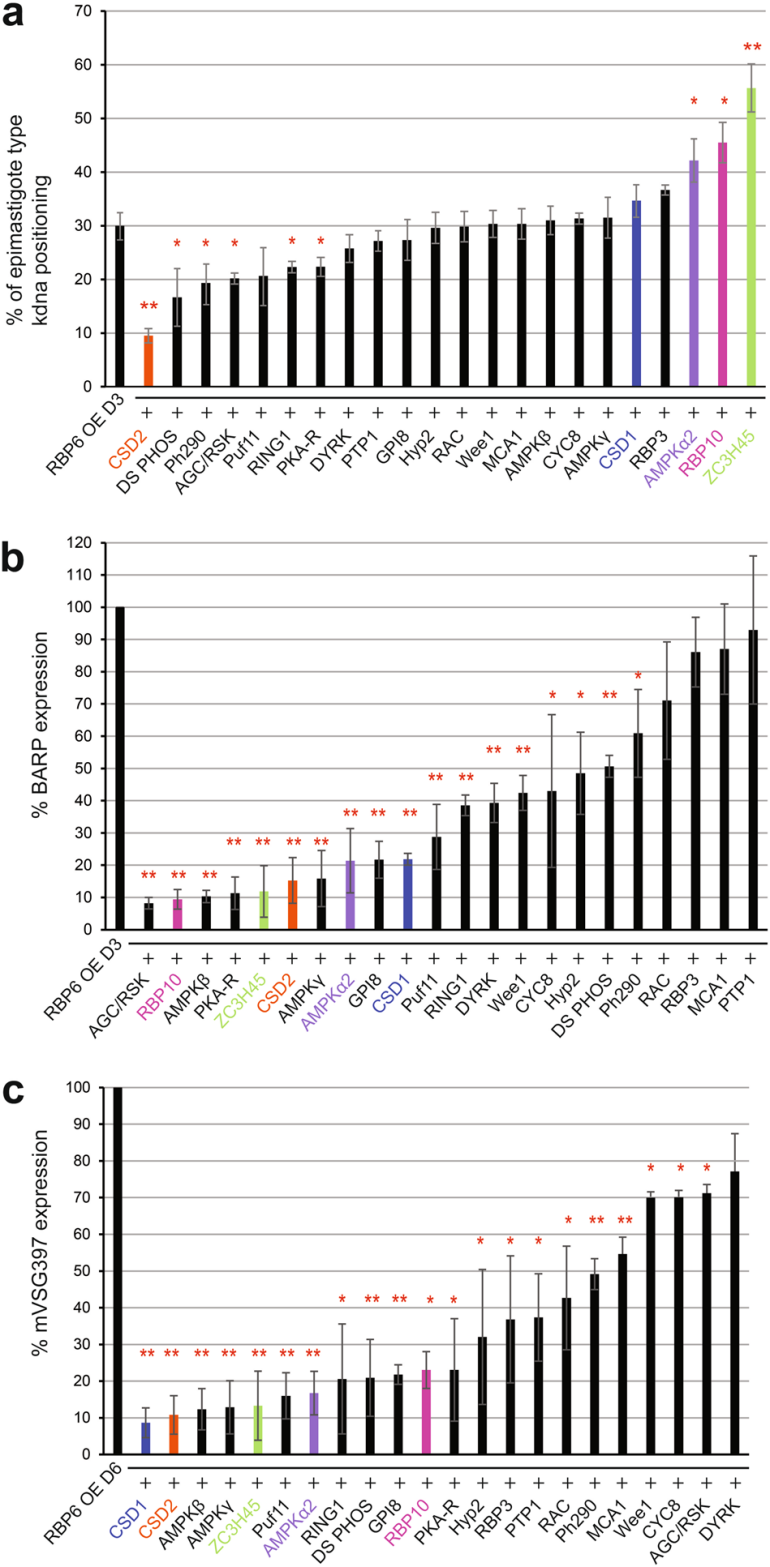
The targeted RNAi screen identified 22 genes that positively or negatively regulate a distinct step of metacyclogenesis. (**a**) After 3 days of doxycycline induction, epimastigotes were scored if the kinetoplast localized anterior to the nucleus in the positive control, the wild-type RBP6 overexpression cell line, and RNAi cell lines. (**b**) Three days post-induction, BARP expression was quantified using Western blot against BARP and compared to the positive control. (**c)** mVSG397 expression was quantified six days post-induction by Western blot against mVSG397. The positive control expression of BARP and mVSG397 is set to 100% and the expression of BARP and mVSG397 in each RNAi cell line is a percentage ratio of the positive control. Three independent replicates were performed with means ± standard deviation (std). The P value was calculated from a two-tailed student’s t-test. *Denotes P values from 0.05 to 0.01, while **Denotes P values < 0.01. RNAi cell lines that were used for downstream RNA-Seq analysis are color coded.

Since we have previously observed that low levels of RBP6 expression inefficiently stimulate metacyclic development^18^, we first assessed RBP6 expression levels in the generated RNAi cell lines after 24 h of doxycycline induction. In comparison to the parental RBP6 expressing cell line, each of the RNAi cell lines expressed RBP6 at a similar level (Supplementary Figs. S2 and S3). Next, we assessed whether RNAi affected the morphological appearance of epimastigote parasites. The most severe phenotype we observed was caused by the down-regulation of cold shock domain-containing protein 2 (CSD2; Tb927.4.4520) with a 66% reduction in the number of epimastigote parasites, relative to the RBP6 overexpression cell line (Fig. 3a). Additionally, in 5 RNAi cell lines we observed a slight, but statistically significant effect on the morphological appearance of epimastigotes (Fig. 3a). The 5 genes responsible for this phenotype encode a dual-specificity phosphatase (DS PHOS, Tb927.7.7160), a phosphatase and tensin homolog (Ph290, Tb927.11.290), a AGC/RSK family kinase (AGC/RSK, Tb927.3.2690), a ring finger domain containing protein (RING1, Tb927.7.6190), and the protein kinase A-regulatory subunit (PKA-R, Tb927.11.4610). Interestingly, the down-regulation of AMP-activated protein kinase α2 subunit (AMPKα2, Tb927.3.4560), RBP10 (Tb927.8.2780), and zinc finger domain containing protein 45 (ZC3H45, Tb927.11.8470) increased the appearance of epimastigote parasites by at least 40% relative to the parental strain. The specificity of the RNAi constructs against AMPKα2 and AMPKα1 was confirmed by the differential abundance of the phosphorylated form of the proteins during the induction (Supplementary Fig. S4). Taken together, we identified 6 positive and 3 negative effectors of kinetoplast repositioning in the initial phase of the developmental program.

Next, we evaluated by Western blotting which of the 74 RNAi cell lines affected BARP expression after three days of RBP6 overexpression. The RNAi-induced down-regulation of 18 individual transcripts resulted in a statistically significant decrease in BARP expression relative to the 3-day induced RBP6 overexpression cell line (Fig. 3b, Supplementary Fig. S5 and Supplementary Table S3). Among these 18 genes were the 9 RNAi cell lines we have shown above to positively or negatively affect the morphological appearance of epimastigotes. In addition, we identified 9 cell lines that displayed normal kinetoplast repositioning, but BARP expression was decreased by approximately 40% (Fig. 3b, Supplementary Fig. S5 and Supplementary Table S3). These 8 genes code for a GPI-anchor transamidase subunit 8 (GPI8, Tb927.10.13860), a Pumillio/PUF RNA-binding protein 11 (Puf11, Tb927.11.10810), a hypothetical protein 2 (Hyp2, Tb927.9.4080), an AMP-activated protein kinase β subunit (AMPKβ, Tb927.8.2450), an AMP-activated protein kinase γ subunit (AMPKγ, Tb927.10.3700), a cold shock domain-containing protein 1 (CSD1, Tb927.8.7820), a cyclin 8 (CYC8, Tb927.7.1390), a Wee1-like protein kinase (Wee1, Tb927.4.3420), and a DYRK protein kinase (DYRK, Tb927.10.15020).

Finally, we monitored each of the 74 RNAi cell lines by Western blotting for their ability to express mVSG397 after 6 days of RBP6 overexpression (Fig. 3c, Supplementary Fig. S5 and Supplementary Table S3). We identified at total of 18 cell lines that displayed at least a 50% reduction in mVSG397 expression, when compared to the 6-day induced RBP6 overexpression cell line. Downregulation of AGC/RSK, CYC8, and Wee1 only resulted in a slight, but statistically significant decrease in mVSG397 expression (Fig. 3c, Supplementary Fig. S5 and Supplementary Table S3). In contrast, RNAi reduction of the remaining identified 14 transcripts that affected epimastigote kinetoplast repositioning and/or BARP expression impaired mVSG397 expression up to 90%. This final phenotypic analysis also revealed four cell lines with normal kinetoplast repositioning and BARP expression, but decreased mVSG397 expression. The four targeted transcripts encode RNA binding protein 3 (RBP3, Tb927.11.530), protein tyrosine phosphatase 1 (PTP1, Tb927.10.6690), a rac serine-threonine kinase (RAC, Tb927.6.2550), and a metacaspase (MCA1, Tb927.11.3220), and these proteins are likely to play a role in the later step(s) of the developmental progression to metacyclics. Unexpectedly, we noticed that the down-regulation of DYRK, which displayed normal kinetoplast repositioning (Fig. 3a and Supplementary Table S3), but reduced BARP expression (Fig. 3b, Supplementary Fig. S5 and Supplementary Table S3), did not result in a significant reduction of mVSG397 expression.

### RNA-seq analysis of selected cell lines

**Figure 4:**
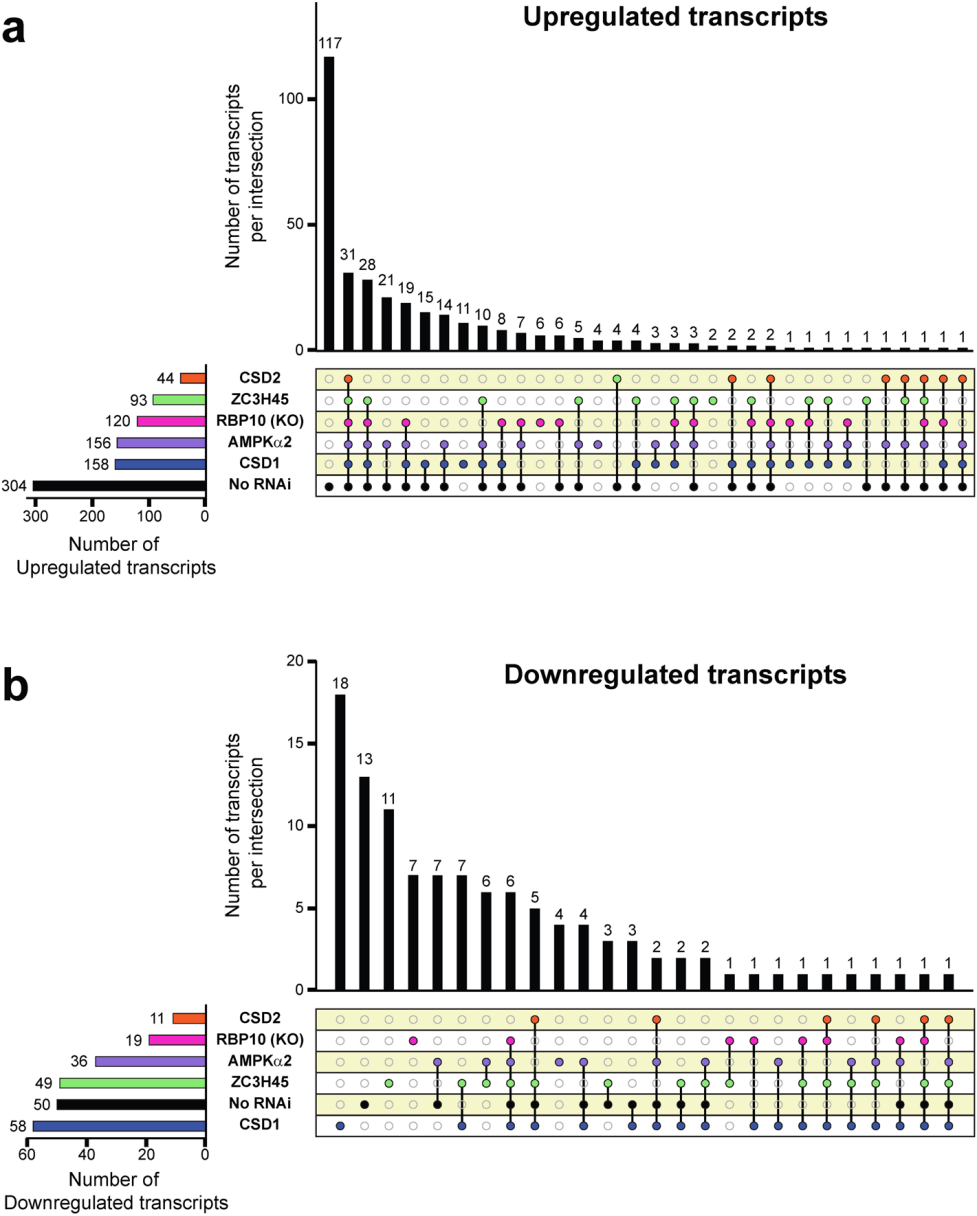
Differentially expressed genes shared across selected RNAi cell lines and RBP10 KO cells in the background of the RBP6 expression at day 1 and 2 after induction. (**a**) UpSet^48^ visualization of upregulated genes. While each row of the combination matrix shows a set of all upregulated genes per experimental (RBP10 KO or RNAi against AMPKα2, CSD1, CSD2 and ZC3H45) and control (No RNAi) groups, columns correspond to intersections, meaning upregulated genes shared between sets. The upper bar graph shows the number of upregulated genes per intersection, while the lateral bar graph shows the number of upregulated genes per set. If a set is participating in an intersection, the corresponding matrix cell is filled with a distinctive color and a black line connecting the participating intersections is displayed (e.g., 31 genes are upregulated in all the compared sets). Upregulated genes found only in one set are depicted with filled cells but no connecting lines (e.g., 117 genes were upregulated exclusively in the “No RNAi” control group). (**b**) UpSet^48^ visualization of downregulated genes. Legends as in panel (**a**) but showing the corresponding analysis for downregulated genes. Website link: https://gehlenborglab.shinyapps.io/upsetr/.

**Figure 5:**
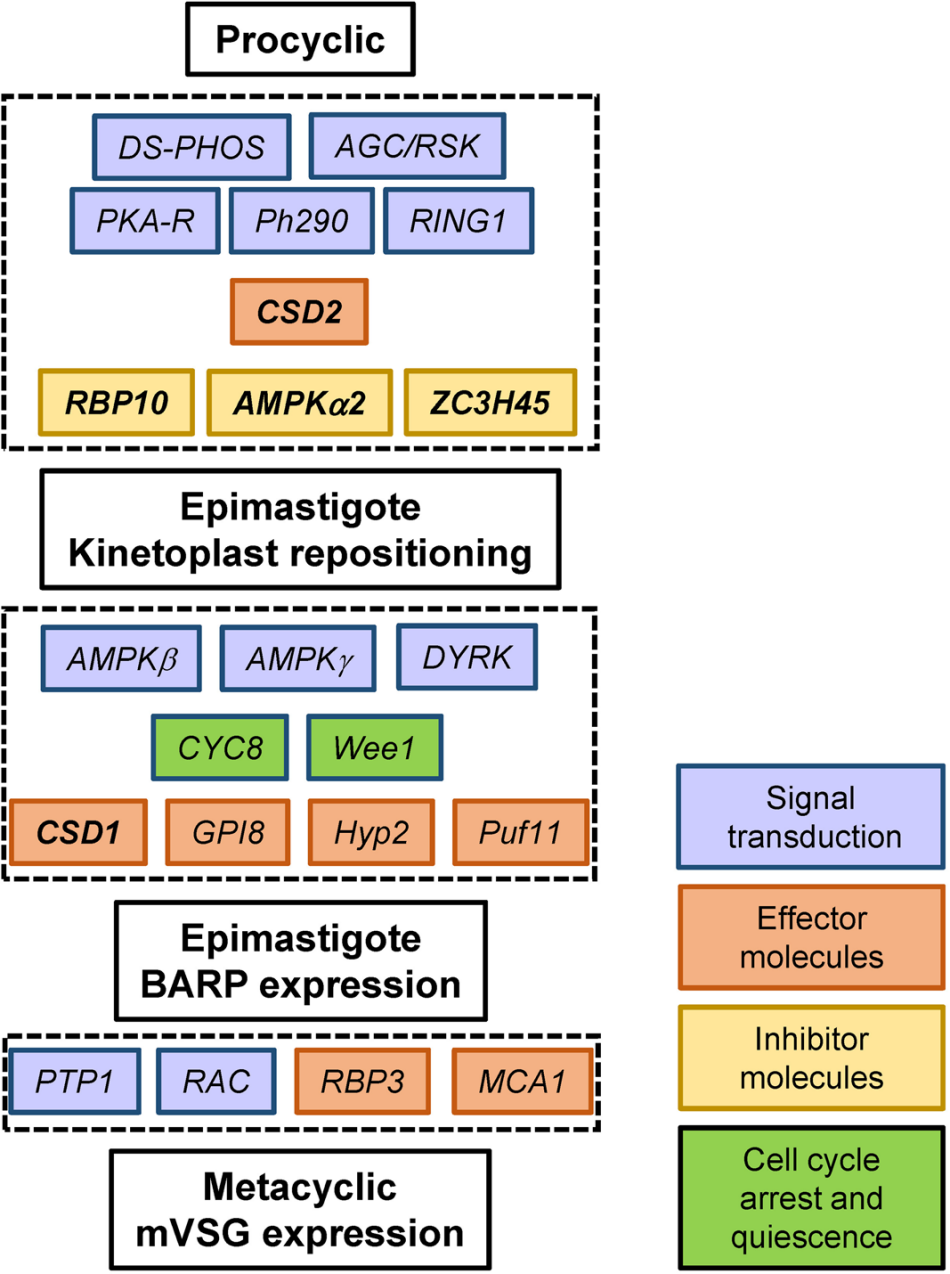
Schematic of identified components regulating metacyclogenesis in *T. brucei*. The order of components at each step and the position of pathway inhibitors is unknown. Those in bold italics were analyzed by RNA-Seq.

The targeted RNAi screen described above identified 22 genes, which are required at different steps in the development of infectious metacyclics (Fig. 5 and Supplementary Table S3). To begin to gain an understanding of the specific role(s) these genes play, we concentrated on RNA/DNA binding proteins and a signal transducer and performed RNA-Seq on the RNAi cell lines of AMPKα2, ZC3H45, CSD1, and CSD2, and the RBP10 KO cell line. It is important to note that the initial phenotypic analysis for RBP10 was done with the RNAi cell line. The subsequent establishment of an RBP10 KO cell line in the RBP6 overexpression background confirmed the RNAi results (Supplementary Tables S4 and S5) and thus we used the KO cell line in subsequent analyses. Three biological replicates of un-induced, day 1-, 2-, 3-, 4- and 6-induced cells were processed (Supplementary Tables S6, S7, S8, S9 and S10), and their transcriptomes were compared to the parental RBP6 over-expressing cell line. To minimize potential secondary effects of RNAi-induced down-regulation, we restricted the analysis reported here to days 1 and 2. During this time period overexpression of RBP6 revealed 304 transcripts with a more than two-fold higher abundance, as compared to un-induced cells, and 50 transcript had a lower abundance^19^. In our initial screen, CSD2 down-regulation displayed the most severe phenotype affecting the early repositioning of the kinetoplast (Fig. 3a) and this was reflected in the RNA-Seq data with only 44 and 11 transcripts having a higher and lower abundance, respectively (Fig. 4 and Supplementary Table S11). Most interestingly, during the first two days the abundance of 5 mVSG transcripts (mVSG397, mVSG653, mVSG1954, mVSG531, mVSG639) increased to levels comparable to the parental RBP6 cell line, indicating that CSD2 does not play a major direct role in mVSG activation. In contrast, in the RBP10 KO cell line the 5 mVSG transcripts were downregulated (Supplementary Table S12), indicating that RBP10 may have a role in stabilizing mVSG transcripts. The mVSG abundance did not change in the CSD1 (Supplementary Table S13), AMPKα2 (Supplementary Table S14) and ZC3H45 RNAi cells (Supplementary Table S15) when compared to un-induced cells, suggesting that these three proteins play a role in mVSG activation directly or indirectly.

There are several additional conclusions we can draw from the RNA-Seq data. The effect on the landscape of upregulated transcripts by the various RNAi cell lines was largely restricted to transcripts changing in the parental cell line, i.e. a very limited number of additional transcripts were upregulated (Fig. 4a) and the number of mRNAs uniquely upregulated in individual RNAi cell lines was surprisingly low (Table 1). In contrast, the downregulated transcripts revealed potential new targets of regulation, including the mVSGs discussed above, since they were only downregulated in the RNAi cell lines (Fig. 4b and Table 2). Of particular interest is the down-regulation of two families of proteins containing META domains in the ZC3H45 RNAi cell line (Supplementary Table S15). These gene families are clustered at the beginning of a polycistronic transcription unit on chromosome 5 and encode either a 446 aa-long protein with two predicted META domains (Tb927.5.2190, 2220, 2250) or a 116 aa-long protein with a single predicted META domain (Tb927.5.2160, 2170, 2200, 2230, 2260). The META domain of about 100 aa has been described in secreted bacterial proteins that are implicated in motility^35–37^. In addition, the syntenic protein meta-1 in *Leishmania major* was localized in the flagellar pocket^38^, was mainly expressed in infective metacyclics^38,39^ and overexpression in *L. amazonensis* increased virulence^40^. The abundance of the *T. brucei* transcripts of both families did not change during metacyclogenesis^19^, but the transcripts were significantly downregulated in purified metacyclics as compared to procyclics (between 8.6- and 11-fold^23^), which was also evident at the protein level^23^, suggesting that ZC3H45 has a role in stabilizing these transcripts during metacyclogenesis.

**Table 1:** Uniquely upregulated transcripts in individual RNAi and KO cell lines.

Cell line	Gene ID	Product description	Gene name
AMPKα2	Tb927.1.5060	Variant surface glycoprotein (VSG)-related, putative	Null
	Tb927.11.11740	Membrane-bound acid phosphatase, putative	Null
	Tb927.11.2400	Flabarin-like protein	Null
	Tb927.5.4000	Hypothetical protein	Null
CSD1	Tb927.10.11970	Glutamine aminotransferase (GlnAT)	GlnAT
	Tb927.10.12580	Hypothetical protein	Null
	Tb927.10.4050	Serine palmitoyltransferase, putative	Null
	Tb927.10.4350	Hypothetical protein	Null
	Tb927.2.4200	CMGC/CLK family protein kinase, putative	Null
	Tb927.4.2410	Glycosyl hydrolase family 65 central catalytic domain/Haloacid dehalogenase-like hydrolase, putative	Null
	Tb927.6.370	Hypothetical protein, conserved	Null
	Tb927.6.550	Hypothetical protein	Null
	Tb927.7.4110	Kinesin, putative	Null
	Tb927.7.6330	Hypothetical protein	Null
	Tb927.8.5350	Hypothetical protein, conserved	Null
RBP10 (KO)	Tb927.10.2500	Hypothetical protein, conserved	Null
	Tb927.11.11400	Hypothetical protein	Null
	Tb927.7.1940	Hypothetical protein	Null
	Tb927.7.3250	Expression site-associated gene 6 (ESAG6) protein, putative	Null
	Tb927.7.3260	Expression site-associated gene 7 (ESAG7) protein, putative	Null
	Tb927.9.9370	PSP1 C-terminal conserved region, putative	Null
ZC3H45	Tb927.1.3710	Hypothetical protein, conserved	Null
	Tb927.11.900	Isocitrate dehydrogenase, putative	IDH

**Table 2:** Uniquely downregulated transcripts in individual RNAi and KO cell lines.

Cell line	Gene ID	Product description	Gene name
AMPKα2	Tb927.1.4970	Hypothetical protein	Null
	Tb927.11.17040	Procyclic-enriched flagellar receptor adenylate cyclase 1	ACP1
	Tb927.6.2160	Hypothetical protein, conserved	Null
	Tb927.9.13200	Hypothetical protein	Null
CSD1	Tb927.10.10010	mRNA turnover protein 4 homolog, putative	MRT4
	Tb927.10.12080	Hypothetical protein, conserved	Null
	Tb927.10.12170	RAB-interacting protein, putative	Null
	Tb927.10.2450	Hypothetical protein	Null
	Tb927.10.6570	TPR repeat, putative	Null
	Tb927.10.8470	Glucose transporter, putative	THT1-
	Tb927.11.12700	Hypothetical protein, conserved	Null
	Tb927.11.1310	NADH-cytochrome b5 reductase, putative	B5R
	Tb927.11.16130	Nucleoside diphosphate kinase	NDPK
	Tb927.11.3610	Nucleobase/nucleoside transporter 8.1	NT8.1
	Tb927.11.3620	Nucleobase/nucleoside transporter 8.2	NT8.2
	Tb927.11.4440	Hypothetical protein	Null
	Tb927.2.5140	Hypothetical protein, conserved	Null
	Tb927.2.6090	60S ribosomal protein L44	RPL44
	Tb927.5.1460	Possible lysine decarboxylase, putative	Null
	Tb927.5.2180	Hypothetical protein, conserved	Null
	Tb927.8.2080	Fungal tRNA ligase phosphodiesterase domain containing protein, putative	Null
	Tb927.9.12030	Hypothetical protein, conserved	Null
RBP10 (KO)	Tb927.11.13740	Procyclic-enriched flagellar receptor adenylate cyclase 5	ACP5
	Tb927.11.18430	Variant surface glycoprotein (VSG), putative	Null
	Tb927.8.7350	Trans-sialidase, putative	Null
	mVSG 1954	Metacyclic variant surface glycoprotein 1954	Null
	mVSG 397	Metacyclic variant surface glycoprotein 397	Null
	mVSG 531	Metacyclic variant surface glycoprotein 531	Null
	mVSG 639	Metacyclic variant surface glycoprotein 639	Null
ZC3H45	Tb927.10.12780	Zinc finger CCCH domain-containing protein 37	ZC3H37
	Tb927.10.8500	Glucose transporter, putative	THT2-
	Tb927.10.9550	Hypothetical protein	Null
	Tb927.4.1560	Hypothetical protein, conserved	Null
	Tb927.5.2190	META domain/Domain of unknown function (DUF1935), putative	Null
	Tb927.5.2220	META domain/Domain of unknown function (DUF1935), putative	Null
	Tb927.5.2230	Conserved protein	Null
	Tb927.5.2240	Hypothetical protein, conserved	Null
	Tb927.5.2250	META domain/Domain of unknown function (DUF1935), putative	Null
	Tb927.5.3320	Pseudokinase, putative	Null
	Tb927.9.14940	SLACS reverse transcriptase, putative	Null

In addition, in the CSD1 RNAi cell line 58 transcripts were downregulated and this included 29 novel transcripts that are potentially regulated by CSD1 through mRNA stability (Supplementary Table S13). The analysis of the coding region and the 3′ UTR of these 29 transcripts by MEME (Multiple Em for Motif Elicitation) identified a purine-rich element (RARGARRAKV), but further analysis will be required to investigate a potential interaction of CSD1 with these transcripts. The motif did not match any previously identified sequences involved in the developmental regulation of *T. brucei* genes and Gene Ontology analysis did not reveal any significant enrichment. Finally, the differentially regulated transcripts that appeared multiple times across the RNAi and KO cell lines (Fig. 4) did not expose specific gene expression regulatory networks.

## Discussion

Developmental progression to infectious metacyclic trypanosomes in the tsetse fly is a critical factor for the transmission of African sleeping sickness and nagana. We gained insight into the regulation of this process with a targeted RNAi screen which revealed genes governing different steps of the metacyclogenesis pathway. Both positive and negative regulators emerged from our assays, and additionally, genes involved in controlling the transition of bloodstream forms to stumpy forms, as well as the differentiation from stumpy to procyclic forms had an effect on metacyclic cell production. Thus, the generation of metacyclics relies on a combination of factors that are required for two distinct processes in bloodstream form trypanosome biology: (i) transition to quiescence and (ii) differentiation from bloodstream to procyclic forms. The likely explanation for this observation is that in addition to establishment of quiescence^23^, metacyclic cells also thoroughly remodel their surface with the synthesis of a VSG coat and their transcriptome largely resembles the bloodstream form transcriptome^23,41^.

The dominant group of genes that exhibited effects on metacyclogenesis in our RNAi screen were signal transducers (Fig. 5). DYRK, PKA-R, AMPKα2, AMPKβ, AMPKγ protein kinases and DS-PHOS protein phosphatase have been implicated in quorum sensing during the development of stumpy bloodstream forms^3^ and all had a pronounced effect on the generation of metacyclics. Genes that have been shown to regulate bloodstream to procyclic form differentiation were also represented by signal transducers and effectors. The protein phosphatase PTP1^42^, a key component of the signaling cascade during this transition, had an effect on mVSG coat synthesis, whereas RBP10, a protein largely responsible for the establishment and maintenance of bloodstream-form-type transcriptome^20–22^ and a strong inhibitor of the bloodstream to procyclic differentiation^20–22^, did exhibit a positive effect on metacyclogenesis. Additionally, our RNA-Seq data revealed that repressor of differentiation kinase 2 (RDK2) was affected in all of the analyzed RNAi cell lines (Supplementary Table S16). This kinase has been identified as a critical regulator of the differentiation from bloodstream to procyclic forms^43^ and it is the most upregulated kinase in metacyclics compared to procyclics^23^.

Two previously uncharacterized putative nucleic acid binding proteins emerged as potent regulators of metacyclogenesis. The cold shock domain-containing proteins CSD1 and CSD2 exhibited very strong effects on metacyclogenesis upon RNAi knockdown, albeit at different steps of the process. Additionally, CSD1 RNAi resulted in the largest number of downregulated mRNAs, exposing a number of transcripts potentially regulated by CSD1 through stabilization (Supplementary Table S13).

Mitochondrial metabolism has a pronounced effect on trypanosome development and it was previously shown to affect the type of procyclin on the surface of procyclic cells^44^, as well as the generation of metacyclics in vitro by inducible RBP6 expression^25^. Alternative oxidase channels electrons directly to oxygen and severely limits ATP production by oxidative phosphorylation. It is highly upregulated in both metacyclics and bloodstream forms and it was recently shown that it contributes to the generation of reactive oxygen species (ROS) which play a major role in the control of metacyclic production^25^. Surprisingly, the mRNA for alternative oxidase was not affected in any of the RNAi or KO cell lines for which we performed RNA-Seq and showed normal up-regulation (Table 3). However, metacyclogenesis in these cell lines was diminished greatly. This indicates that ROS may be required, but they are not sufficient to trigger efficient developmental progression. One clear example of a mRNA which was affected in all of the tested RNAi cell lines (Supplementary Table S16) is coding for the mitochondrial malic enzyme (Tb927.11.5450). In *T. brucei* this enzyme is believed to convert l-malate to pyruvate irreversibly and its down-regulation has a strong growth phenotype^45^. As pyruvate is the main end product of metabolism in bloodstream form trypanosomes, this suggests that the balance between L-malate and pyruvate (and/or the balance of NADP+ , the cofactor in the malic enzyme reaction, and NADPH in the mitochondrion) could possibly play an important role in regulating generation of metacyclics in addition to ROS. This is supported by the observation that pyruvate dehydrogenase E1α subunit (Tb927.10.12700) showed fourfold higher phosphorylation in metacyclics relative to procyclics^23^, which in other organisms has been demonstrated to inhibit enzyme function, i.e. pyruvate is blocked from entering the TCA cycle and it is likely to be the end product of metabolism in developing metacyclics.

**Table 3:** Transcripts not affected in any of the RNAi and KO cell lines.

Gene ID	Product description	Gene name
Tb927.10.7090	Alternative oxidase, mitochondrial	AOX
Tb927.4.3990	Amino acid transporter, putative	Null
Tb927.6.5020	Cyclin 7, putative	CYC7
Tb927.10.10770	Generative cell specific 1 protein, putative	GCS1
Tb927.10.13410	Hypothetical protein	Null
Tb927.2.1290	Hypothetical protein	Null
Tb927.4.140	Hypothetical protein	Null
Tb927.6.110	Hypothetical protein	Null
Tb927.7.7560	Hypothetical protein	Null
Tb927.10.14900	Hypothetical protein, conserved	Null
Tb927.2.5290	Hypothetical protein, conserved	Null
Tb927.2.5300	Hypothetical protein, conserved	Null
Tb927.2.5320	Hypothetical protein, conserved	Null
Tb927.2.5350	Hypothetical protein, conserved	Null
Tb927.2.5360	Hypothetical protein, conserved	Null
Tb927.10.13400	Phosphatidic acid phosphatase, putative	Null
Tb927.2.5530	Present in the outer mitochondrial membrane proteome 22–1	POMP22-1
Tb927.2.5610	Present in the outer mitochondrial membrane proteome 22B	POMP22B
Tb927.7.5960	Protein associated with differentiation 4, putative	PAD4
Tb927.7.5980	Protein associated with differentiation 6, putative	PAD6
Tb927.7.6000	Protein associated with differentiation 8, putative	PAD8
Tb927.2.160	Protein of unknown function (DUF1181), putative	Null
Tb927.6.220	purine nucleoside transporter	TbNT9
Tb927.4.300	RETROTRANSPOSON HOT SPOT PROTEIN 3 (RHS3), PUTATIVE	RHS3
Tb927.1.70	RETROTRANSPOSON HOT SPOT PROTEIN 4 (RHS4), PUTATIVE	RHS4
Tb927.2.240	RETROTRANSPOSON HOT SPOT PROTEIN 5 (RHS5), PUTATIVE	RHS5
Tb927.7.2010	RETROTRANSPOSON HOT SPOT PROTEIN 7 (RHS7), PUTATIVE	RHS7
Tb927.1.2160	Small kinetoplastid calpain-related protein 1–5	SKCRP1-5
Tb927.7.2180	*T. brucei* spp.-specific protein, *T. brucei* spp.-specific protein, putative	Null
Tb927.5.3990	Variant surface glycoprotein (VSG, atypical), putative	Null

Our targeted RNAi screen was focused primarily on nucleic-acid-binding proteins and protein kinases and phosphatases. Interestingly, the RNA-Seq data from selected group of these cell lines revealed that these components of gene expression regulatory networks are able to affect the abundance of a subset of transcripts very similarly. This suggests that different post-transcriptional regulons intersect in a defined pool of mRNAs, which exhibit developmentally regulated changes in expression and are under the direct or indirect control of these different RBPs. It is very likely that most or all of these transcripts are the direct targets for several of the RBPs studied here. Interestingly, among this subset is the mRNA for hnRNP F/H, yet another protein with a role in regulating the trypanosome life cycle. HnRNP F/H is highly upregulated in metacyclic^23^ and bloodstream form parasites^46^ in comparison to procyclics and has been shown to influence the efficiency of pre-mRNA trans-splicing and mRNA stability in a life-cycle-dependent manner^46^.

In conclusion, our data suggest a considerable overlap between the genes that regulate the formation of stumpy bloodstream form trypanosomes and the genes that govern the development of metacyclic form parasites. This indicates that the vast majority of these gene expression regulators affect processes associated with cell-cycle arrest and decrease in biosynthetic capacity in cells transitioning to quiescence, a state of *T. brucei* development strongly correlated with transmission in either direction between the tsetse fly vector and the mammalian host.

## Methods

### Polysome analysis and RNA-seq

The RBP6 overexpression cell line^18^ was induced for 48 h with doxycycline at a final concentration of 10 μg/μl. Un-induced and 48 h induced cells were incubated with cycloheximide at a final concentration 0.1 μg/μl for 1 h. Cytoplasmic extracts were prepared using detergent lysis and manual homogenization with polysome buffer (120 mM KCl, 20 mM Tris, pH 7.4, 2 mM MgCl_2_, 1 mM dithiothreitol, 10 μg/ml leupeptin and 0.1 μg/μl cycloheximide), containing 40 Units of RNase inhibitor and 1.2% Nonidet P-40. The cellular lysates were cleared by centrifugation at 14,000 rpm for 4 min. The concentration of nucleic acid in the lysate was measured by absorbance at 260 nm. The supernatants corresponding to 80 OD_260_ were layered onto 15–50% linear sucrose gradients with 0.1 μg/μl cycloheximide in polysome buffer and centrifuged for 2 h at 36,000 rpm in a Beckman SW-41 rotor at 4 °C. The A_254_ profile was recorded using the ISCO UA-6 detector. Fractions of 500 μl were manually collected from the gradient and prior to RNA extraction, 5 ng of in vitro transcribed firefly luciferase (Promega) were added to each fraction to allow for normalization. Each fraction was supplemented with 200 μg/ml of proteinase K, 1% SDS, 10 mM EDTA and incubated at 65 °C for 30 min, followed by RNA isolation using the phenol–chloroform method. Based on the sucrose gradient profile, fractions corresponding to monosomes and those with heavy polyribosomes were pooled separately. Three biological replicates were processed by RNA-Seq.

### Plasmid constructions

To obtain inducible hairpin RNAi constructs, we followed the single-cloning-step procedure for generating RNAi plasmids^33^. First, we created a suitable RNAi vector. The phleomycin resistant construct that integrates at the 177-bp repeat, pLew100.v5-Ble-177-bp rep, was digested with HindIII and PstI to remove the 3′UTR of aldolase. Two oligonucleotides (“GCATCATCTAGACAATCA” and “ACGTCGTAGTAGATCTGTTAGTTCGA”) were annealed exposing the PstI and HindIII sticky ends. The annealed oligonucleotides were ligated into the digested pLew100.v5 (Ble) plasmid. This plasmid contains three restriction sites that are suitable for the single-cloning-step protocol, which are XbaI, HindIII, and PstI. We called this construct pXHPHP and prepped this vector for ligation by individually digesting it with either XbaI or HindIII. Next, we prepared inserts for our RNAi construct, where a 400 to 600 base pair region of the coding sequence of one gene was PCR amplified. This amplicon contained either a HindIII or XbaI restriction site at the 5′ end, while the 3′ end contained approximately 50 base pairs of random sequences and an EcoRI restriction site. The amplicon was digested with EcoRI and self-ligated to form a stuffer-containing inverted repeat insert. The self-ligated insert was digested with either HindIII or XbaI and ligated into the HindIII or XbaI digested pXHPHP vector. These constructs were confirmed by restriction enzyme digest and DNA sequencing. The construct was linearized with NotI prior to transfection. The entire list of primers used for RNAi plasmid construction is available in Supplementary Table S17.

### *T. brucei* cell culture and transfection

The *T. brucei* Lister 427 (29–13) strain that carries the inducibly-expressed RBP6 (Tb927.3.2930) transgene at the rDNA spacer (pLew100.v5-BSD) was cultured at 28 °C and 5% CO_2_ in Cunningham’s media supplemented with 10% Tet-system approved heat-inactivated Fetal Bovine Serum (FBS) and 2 mM l-glutamine, 100 units/ml penicillin, 100 μg/ml streptomycin, 50 μg/ml gentamicin, 15 μg/ml G418, 50 μg/ml hygromycin B, and 10 μg/ml blasticidin. A total of 1 × 10^8^ of the RBP6 overexpression cells were used to transfect an inducible hairpin RNAi construct. Cells were centrifuged at 3500 RPM for 6 min, washed in Cytomix (20 mM KCl, 0.15 mM CaCl_2_, 10 mM K_2_HPO_4_, 25 mM 4-(2-hydroxyethyl)piperazine-1-ethanesulfonic acid (Hepes), 2 mM ethylenediaminetetraacetic acid (EDTA) and 5 mM MgCl_2_, pH7.6), and resuspended in 500 μl Cytomix. Subsequently, 25 μg of linearized plasmid DNA was mixed with the solution and cells were pulsed twice at 1600 V with a time constant of 0.6 ms using a GenePulser Xcell (BioRad, Hercules, CA, USA). The selective drug, phleomycin, was added to the culture medium 24 h after electroporation at a final concentration of 2.5 μg/ml.

### Scoring epimastigotes and metacyclic cells

After 72 h of doxycycline induction of an RNAi cell line, epimastigote parasites were scored from a mixed population after DNA staining by Hoechst. These quantifications were performed with 4% paraformaldehyde fixed cells and/or live cells. Different trypanosome cell types were determined by their size, shape, and position of the kinetoplast relative to the nucleus and the posterior end of the cell.

### Western blot analysis

Western blots were performed as described previously^47^. To stabilize the BARP protein, 0.5 mM of the metalloprotease inhibitor bathophenanthroline disulphonic acid (Acros Organics, catalogue no. AC164050050) was added to each 48-h RBP6 induced cell line and incubated for 24 h. These cells were then harvested for Western blot analysis. Rabbit antiserum against RBP6, BARP, and mVSG397 as well as mouse antiserum against paraflagellar rod (PFR) or elongation factor 1-alpha (EF-1α, clone CBP-KK1 from Sigma-Aldrich) was used at a 1:1000 dilution. Either PFR or EF-1α was used as a loading control for each Western blot. Pierce Protease and Phosphatase Inhibitor Mini Tablets (Thermofisher, catalog no. A32959) was dissolved into the lysis buffer following manufacturer’s instruction and Western blots were probed with the rabbit polyclonal anti-p-AMPK (1:1000) antibody (Phospho-AMPKα (Thr172) (40H9) Rabbit mAb #2535, Cell Signaling technologies). The horseradish peroxidase-conjugated secondary antibody (Roche) was used at a 1:5000 dilution.

### RNA preparation, RNA-Seq, read processing, and data analysis

Total RNA was prepared from approximately 2 × 10^7^ to 5 × 10^7^ un-induced cells, 1-, 2-, 3-, 4-, and 6-day induced cells that express both RBP6, as well as a RNAi hairpin against either AMPKα2, ZC3H45, CSD1, and CSD2, or are KO for RBP10. Three biological replicates were performed at least one week apart, and the RNA was prepared using the TRIzol reagent from Invitrogen according to the manufacturer’s instructions. Isolation of poly(A)^+^ mRNA, library preparation and sequencing on an Illumina HiSeq2500 platform were performed at the Yale Center for Genome Analysis as described previously^19^. Read processing and data analysis was performed as described^19^.

## Supplementary Information

Supplementary Figures.

Supplementary Tables.

## Data Availability

RNA-Seq data from this study have been submitted to the NCBI Sequence Read Archive—SRA at http://www.ncbi.nlm.nih.gov/Traces/sra/sra.cgi with accession numbers: PRJNA657432, PRJNA656316, PRJNA656556, PRJNA657048 and PRJNA657089.
