# Supplementary material for: Identification of positive and negative regulators in the stepwise developmental progression towards infectivity in Trypanosoma brucei
Source: Sci Rep. 2021 Mar 11;11:5755. doi: 10.1038/s41598-021-85225-2 (PMC7952579; doi:10.1038/s41598-021-85225-2)
Supplement: Supplementary file 1 — Supplementary Figures. [file 41598_2021_85225_MOESM1_ESM.pdf]

Identification of positive and negative regulators in the stepwise developmental progression towards infectivity in *Trypanosoma brucei*

Justin Y. Toh, Agathe Nkouawa, Saúl Rojas Sánchez, Huafang Shi, Nikolay G. Kolev, and Christian Tschudi

Department of Epidemiology of Microbial Diseases, Yale School of Public Health, New Haven, CT 06536, USA

Supplementary Figures S1-S8

Supplementary Figure S1. RBP6 Expression for 1 day

Supplementary Figure S2. Quantification of RBP6 levels in RNAi cell lines

Supplementary Figure S3. RBP6 levels in RNAi cell lines full-length blots

Supplementary Figure S4. AMPKα Westerns

Supplementary Figure S5. BARP and mVSG397 Westerns

Supplementary Figure S6. BARP Westerns full-length blots

Supplementary Figure S7. mVSG397 Westerns full-length blots

Supplementary Figure S8. RBP6, BARP and mVSG397 Westerns for RBP10 full-length blots

Supplementary Figure S9. Full-length blots for Fig. 1b and d

Supplementary Figure S10. Full-length blots for Fig. 2

Supplementary Tables S1-S17

Supplementary Table S1. Polysome Data

Supplementary Table S2. RNAi Targets

Supplementary Table S3. Summary RNAi Analysis

Supplementary Table S4. RNA-Seq All Data RBP10 RNAi

Supplementary Table S5. RBP10 RNAi RNA-Seq Data Day 1 and 2

Supplementary Table S6. RNA-Seq All Data CSD1 RNAi

Supplementary Table S7. RNA-Seq All Data CSD2 RNAi

Supplementary Table S8. RNA-Seq All Data ZC3H45 RNAi

Supplementary Table S9. RNA-Seq All Data AMPKα2 RNAi

Supplementary Table S10. RNA-Seq All Data RBP10 KO

Supplementary Table S11. CSD2 RNAi RNA-Seq Data Day 1 and 2

Supplementary Table S12. RBP10 KO RNA-Seq Data Day 1 and 2

Supplementary Table S13. CSD1 RNAi RNA-Seq Data Day 1 and 2

Supplementary Table S14. AMPKα2 RNAi RNA-Seq Data Day1 and 2

Supplementary Table S15. ZC3H45 RNAi RNA-Seq Data Day 1 and 2

Supplementary Table S16. Transcripts affected in all RNAi and KO cell lines

Supplementary Table S17. RNAi oligos

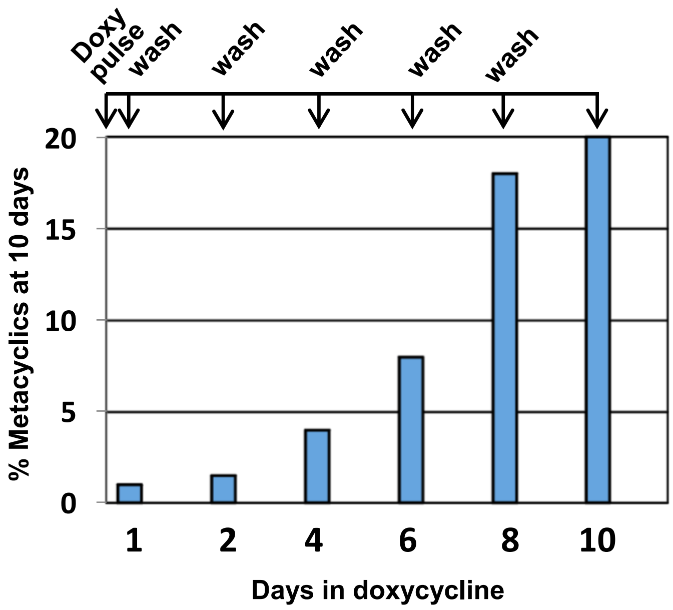

Supplementary Figure S1. RBP6 was induced at day 0 with doxycycline and at the indicated days an aliquot was washed to remove doxycycline and the cells were incubated for up to 10 days. Metacyclics were scored microscopically.

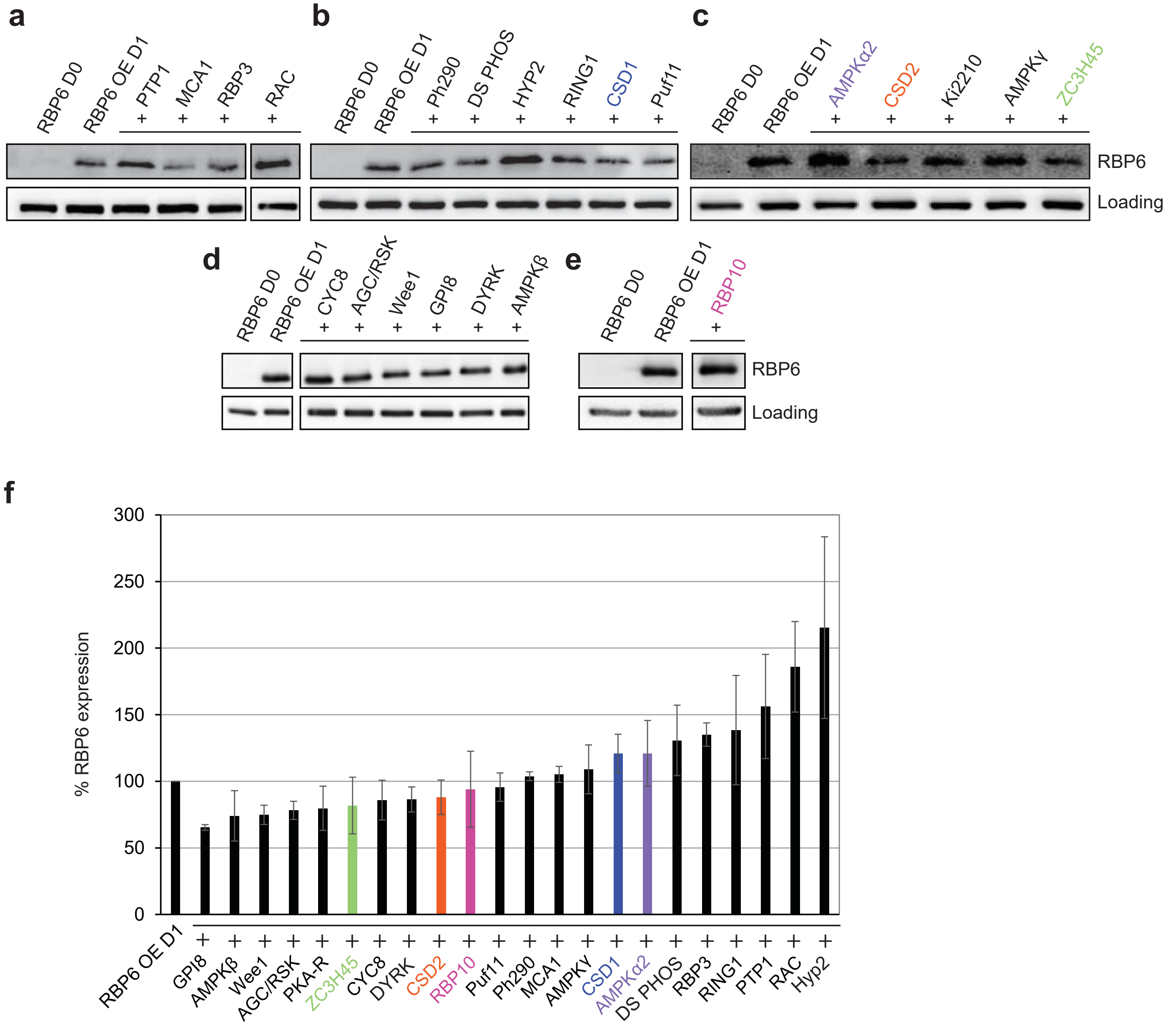

Supplementary Figure S2: Quantification of RBP6 expression in RNAi cell lines.
(a-e) Each RNAi cell line and the positive control, the wild-type RBP6 overexpression cell line, were induced for 1 day and cells were harvested for RBP6 Western blot analysis. Three replicates were performed, and a representative western blot is shown.
(f) The expression of RBP6 from the positive control was set to 100% and the RBP6 expression levels in each RNAi cell line is represented as a percentage ratio of the positive control. The bar graphs depict mean ± standard deviation.
Full-length blots for panels a-d and e are shown in Supplementary Figures S3 and S8, respectively.

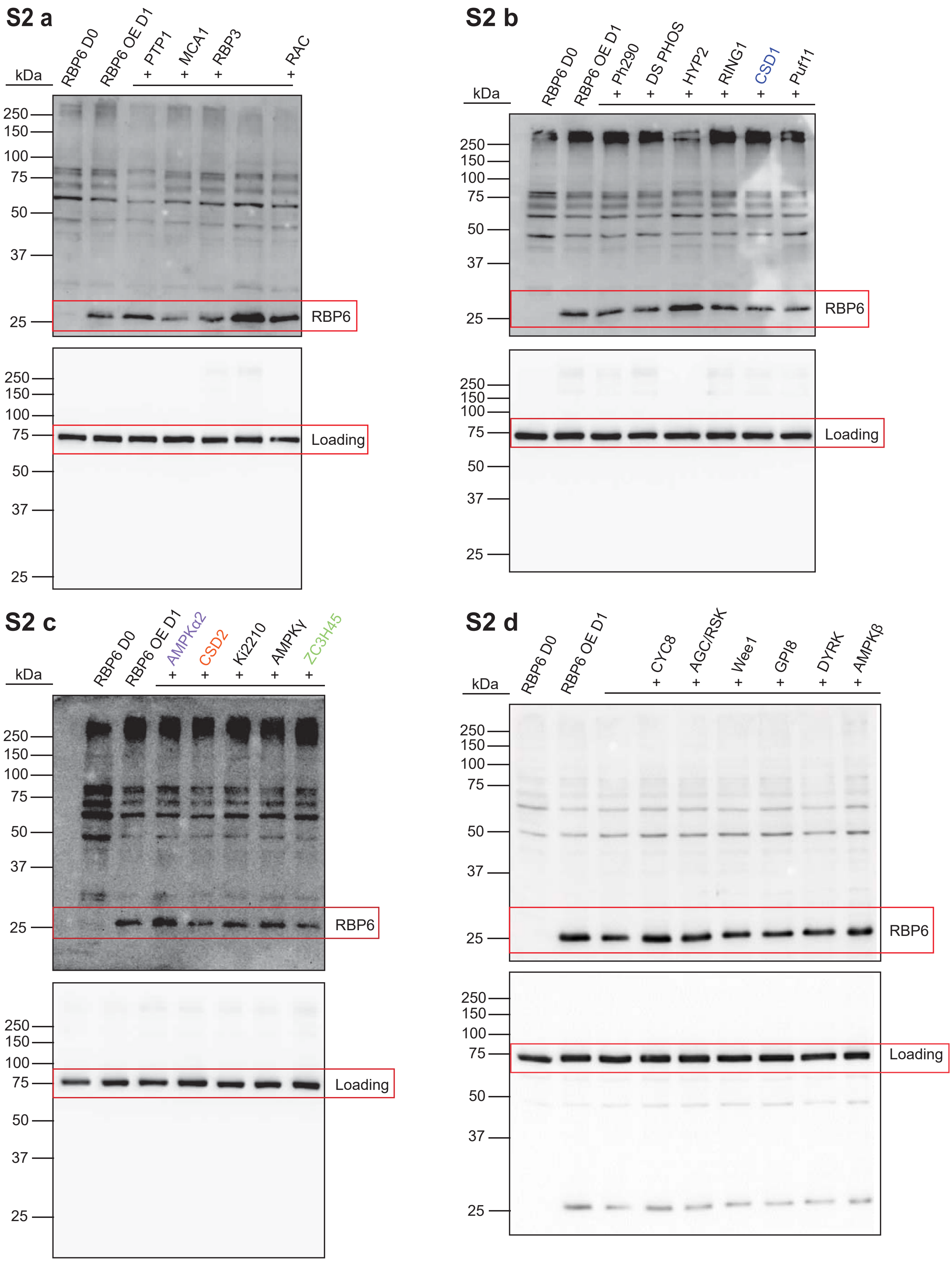

Supplementary Figure S3. Full-length blots of panels a-d in Supplementary Figure S2.
(a) The unlabeled lane between RPB3 and RAC belongs to the RNAi cell line where phosphoglycerate kinase c (PGKC) was depleted in the RBP6 overexpression cell line.
(d) The unlabeled lane between the positive control RBP6 OE D1 and CYC8 belongs to the RNAi cell line where the putative serine/threonine kinase NRKB was downregulated in the RBP6 overexpression cell line.
In this blot, the membrane was first used to probe for RBP6, then the loading control, PFR2.
Red boxes indicate the cropped images shown in Supplementary Figure S2.

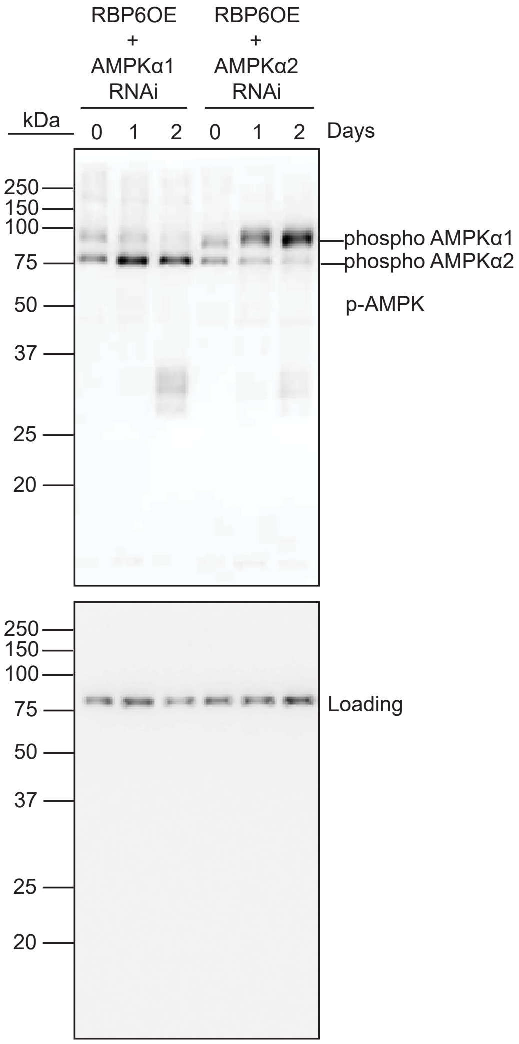

Supplementary Figure S4. Confirming the depletion of either AMPKα1 or AMPKα2 in their respective RNAi cell line using the anti-phospho-Thr172 AMPK antibody. This membrane was first probed for BARP before the anti-phospho-Thr172 AMPK antibody was used. Residual BARP signals remain between the 25 and 37 kDa markers.

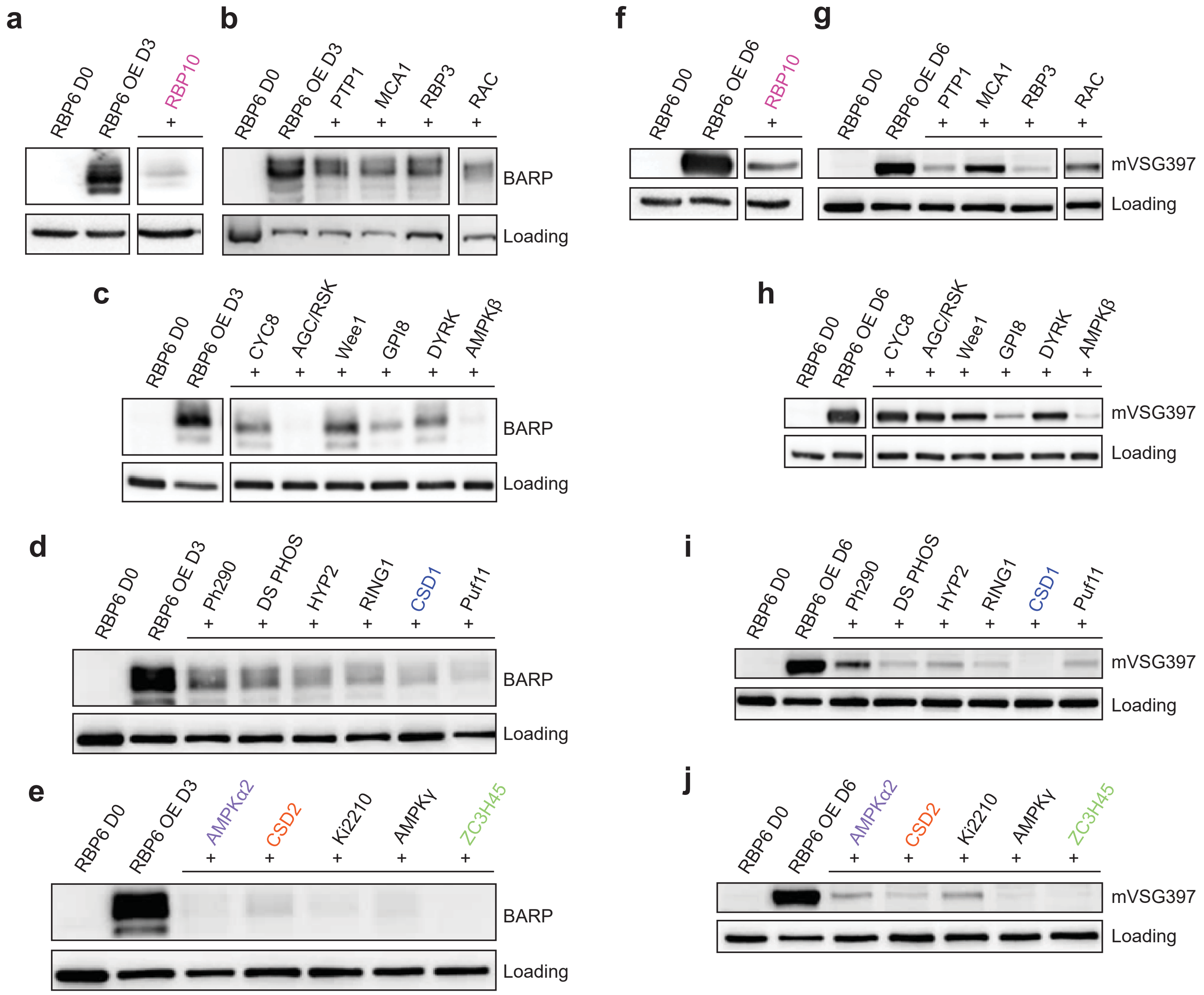

Supplementary Figure S5. Western blot of BARP (a-e) and mVSG397 (f-j) in each RNAi cell line. Representative Western blots against BARP or mVSG397 for each RNAi cell line compared to the induced RBP6 overexpression cell line are shown.
Full-length blots of BARP panels b-e and mVSG panels g-j are shown in Supplementary Figures S6 and S7, respectively.
Full-length blots for RBP10 panels a and f are shown in Supplementary Figure S8.

S5 b

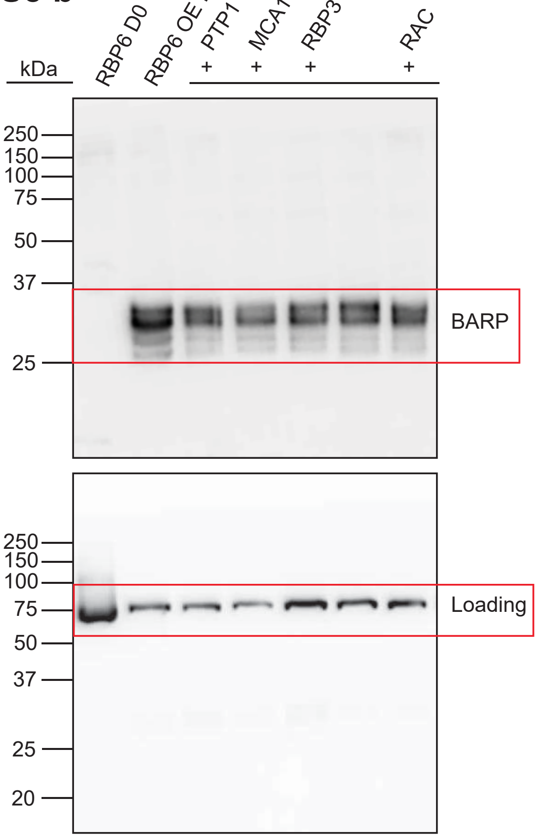

S5 c

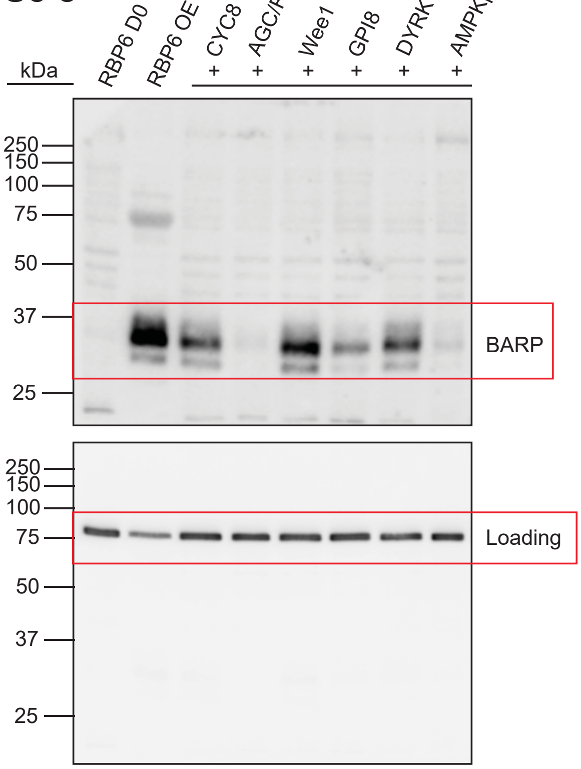

S5 d

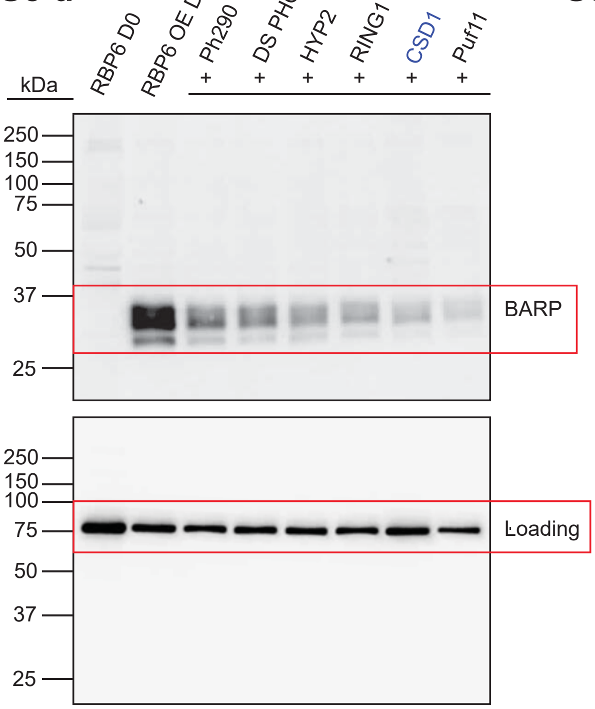

S5 e

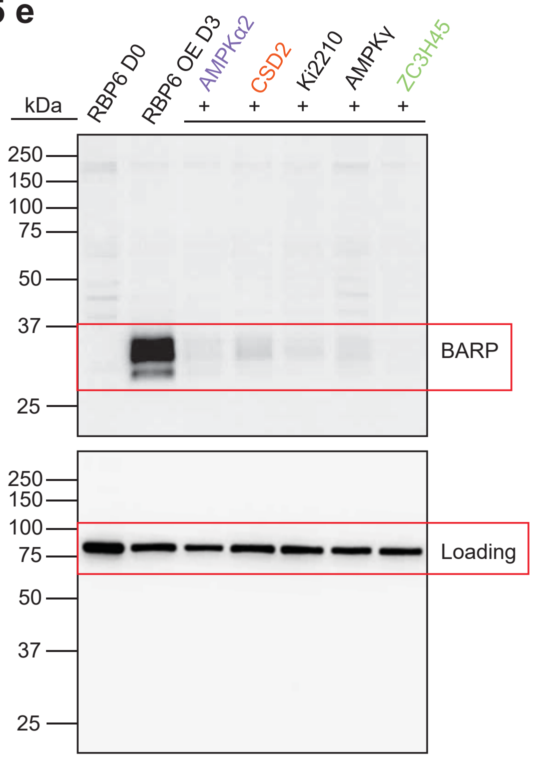

Supplementary Figure S6. Full-length blots of panels b-e in Supplementary Figure S5.
(b) The unlabeled lane between RPB3 and RAC belongs to the RNAi cell line where phosphoglycerate kinase c (PGKC) was depleted in the RBP6 overexpression cell line.
Red boxes indicate the cropped images shown in Supplementary Figure S5.

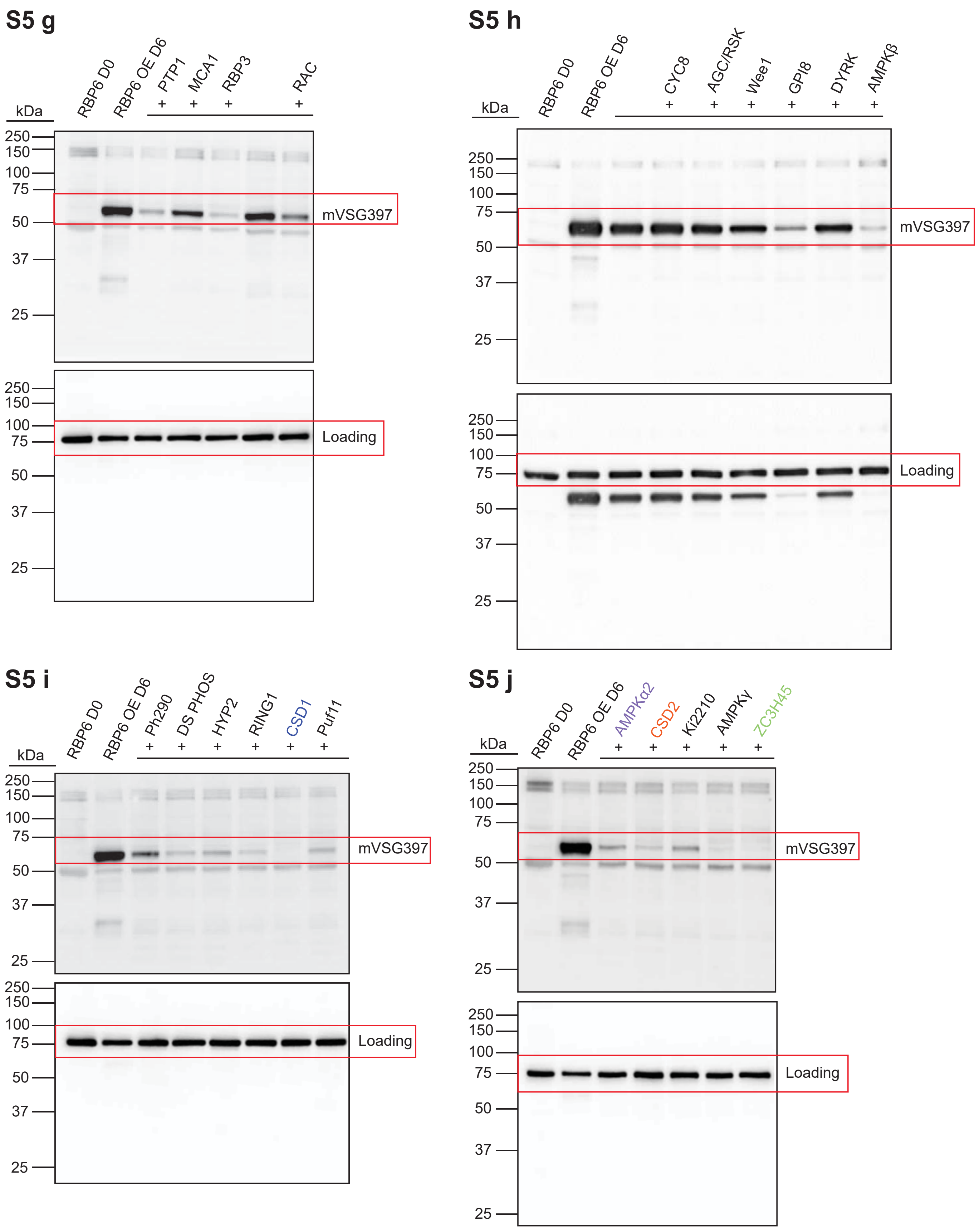

Supplementary Figure S7. Full-length blots of panels g-j in Supplementary Figure S5.
(g) The unlabeled lane between RPB3 and RAC belongs to the RNAi cell line where phosphoglycerate kinase c (PGKC) was depleted in the RBP6 overexpression cell line.
(h) The unlabeled lane between the positive control RBP6 OE D6 and CYC8 belongs to the RNAi cell line where the putative serine/threonine kinase NRKB was downregulated in the RBP6 overexpression cell line. In this blot, the membrane was first used to probe for mVSG397 and subsequently probed for the loading control, PFR2.
The red boxes indicate the cropped images shown in Supplementary Figure S5.

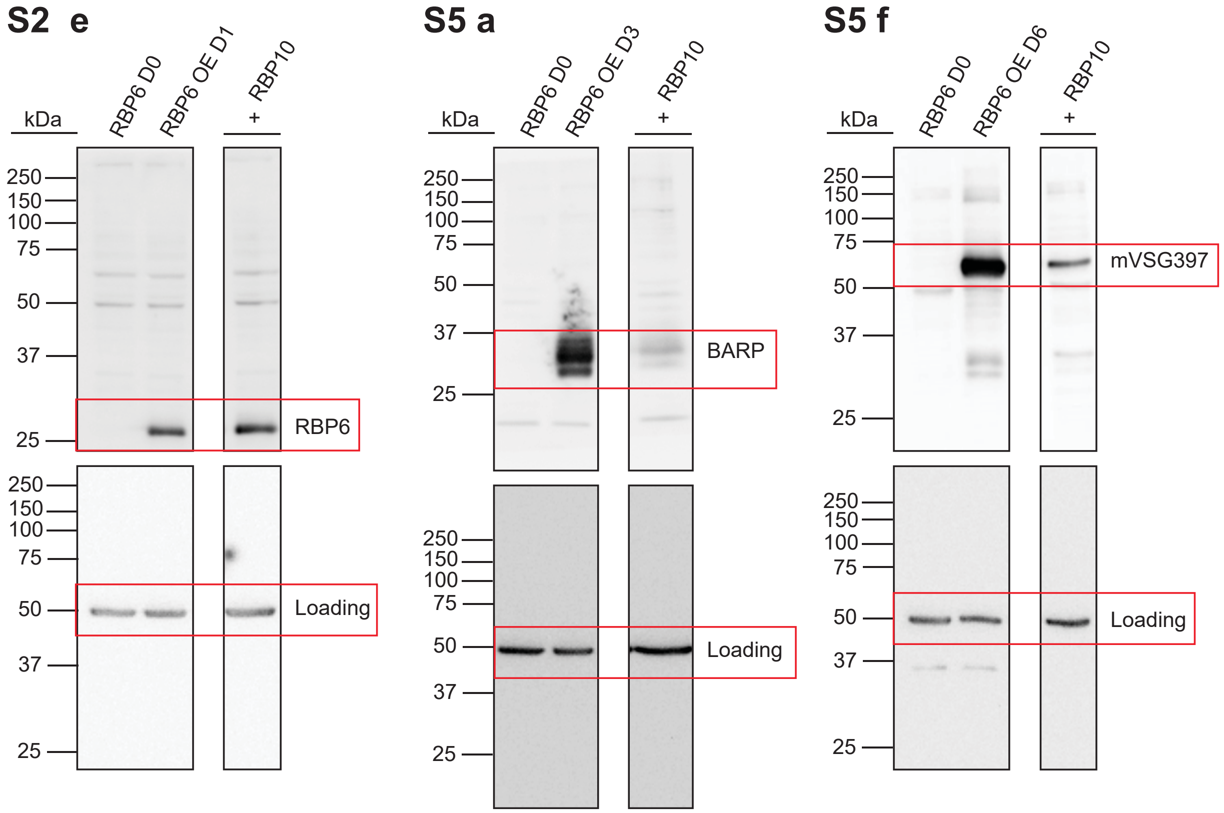

Supplementary Figure S8. Full-length images of RBP6, BARP, and mVSG397 expression in the RBP10 RNAi cell line. The red boxes indicate the cropped images shown in Supplementary Figures S2 and S5.

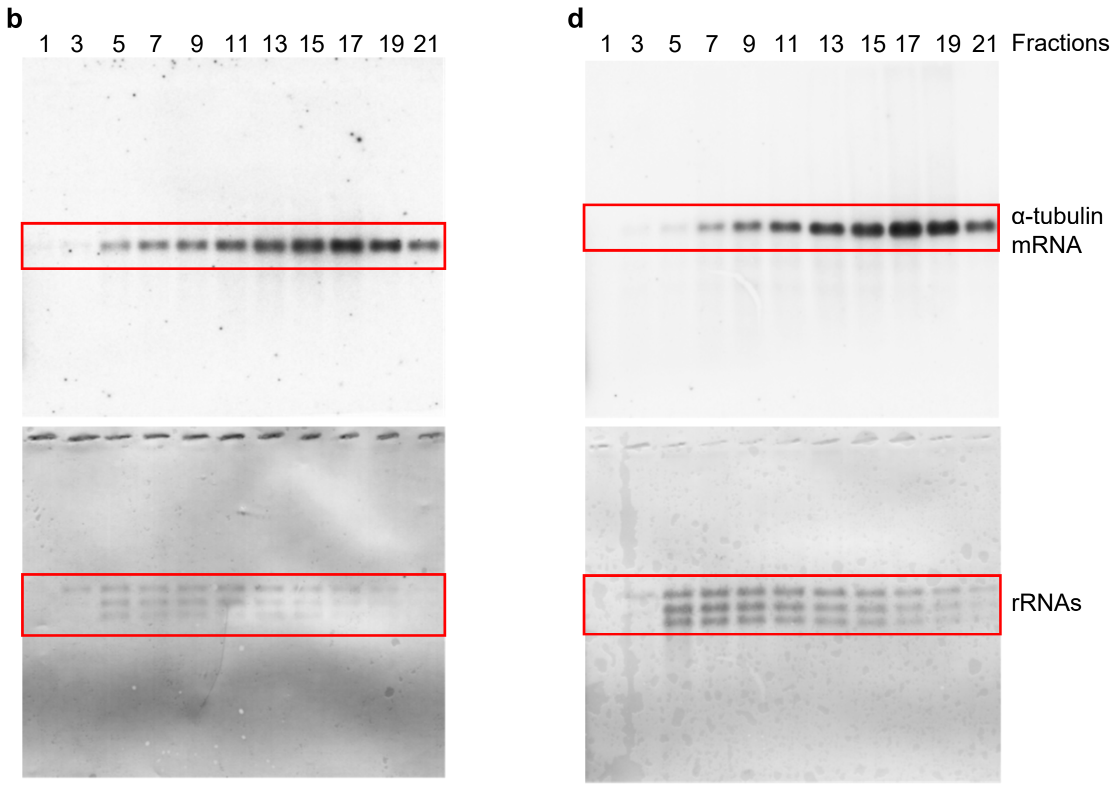

Supplementary Figure S9. Full-length blots of panels b and in Figure 1.
Red boxes indicate the cropped images shown in Figure 1.

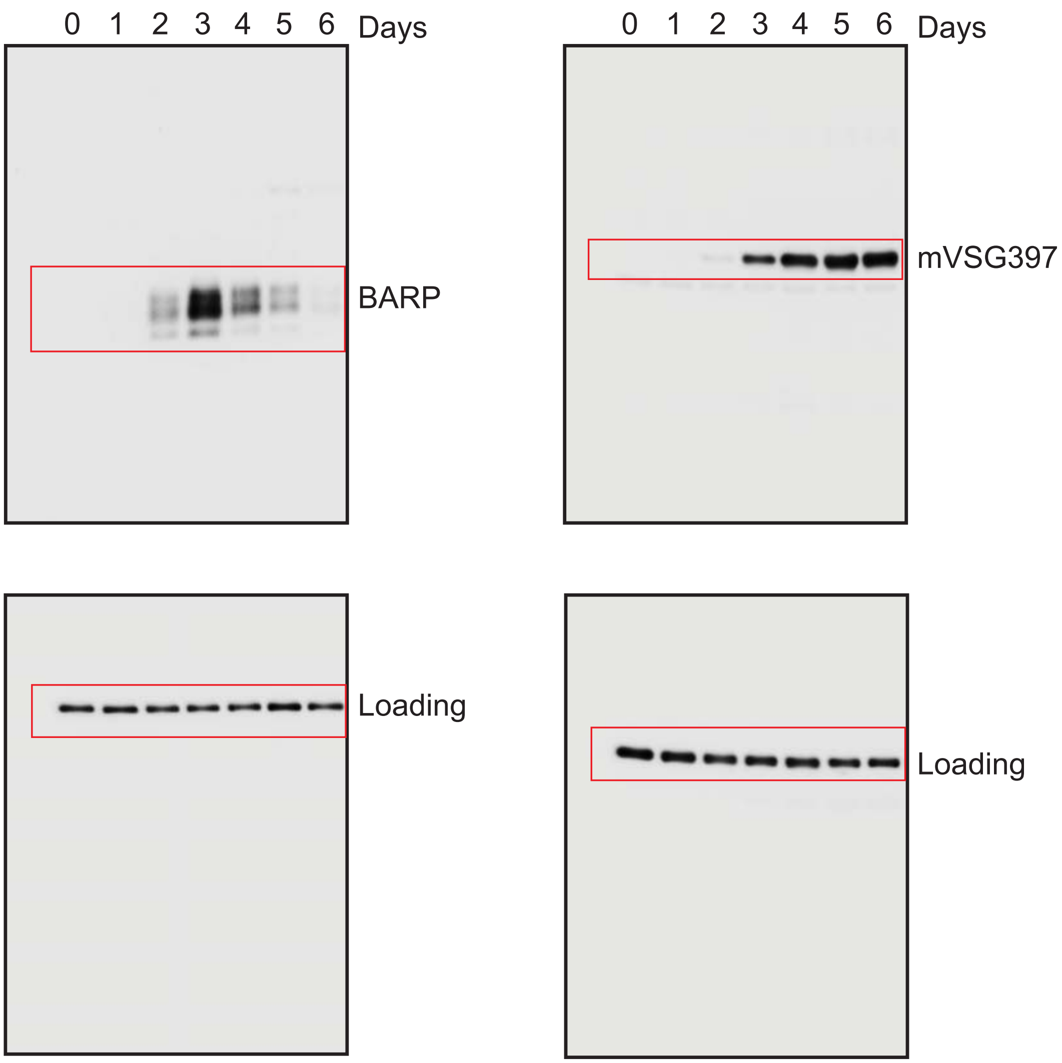

Supplementary Figure S10. Full-length blots of panels shown in Figure 2.
Red boxes indicate the cropped images shown in Figure 2.
